# The outcomes of the computerized training of cognitive functions in patients with MCI in epilepsy

**DOI:** 10.1192/j.eurpsy.2022.634

**Published:** 2022-09-01

**Authors:** I. Blazhina, V. Korostiy

**Affiliations:** 1 Bucovinian State Medical University, Department Of Nervous Diseases Psychiatry And Medical Psychology, Chernivtsi, Ukraine; 2 Kharkiv National Medical University, Department Of Psychiatry, Narcology, Medical Psychology And Social Work, Kharkiv, Ukraine

**Keywords:** Cognitive disorders, MCI, Computerized training, epilepsy

## Abstract

**Introduction:**

Cognitive impairments have a considerable impact on the functioning of patients, their socialization and level of disability. Cognitive deficits significantly deteriorate the quality of patients’ life. Currently, the possibilities of pharmacological correction of cognitive disorders in patients with epilepsy are limited.

**Objectives:**

Study of non-pharmacological program of cognitive disorder correction in patients with epilepsy and the assessment of its efficiency.

**Methods:**

We have studied the features of clinical and psychopathological manifestations in patients suffering from epilepsy. The study included 146 patients with epilepsy (85 men and 61 women) who were receiving inpatient care. The following psychodiagnostic techniques were used: MOCA test, Mini Mult test, Münsterberg test, depression and Hamilton anxiety scale, quality of life scale. 63 patients received cognitive training online, of which 30 patients also used psychoeducation methods.

**Results:**

According to the MoCA findings, patients with epilepsy showed cognitive decline, the average score was 20.72, whereas healthy persons’ average score was 27.36. The Quality of Life Scale: the average rate among all examined persons was 69.45 out of 100, 78.60 were the results of healthy persons. In patients with PG1, who used cognitive training and psychoeducation the results of the MoCA test showed an improvement in cognitive functions (1.4, p <0.001) and increased subjective assessment of quality of life (2.77, p <0.05).

**Conclusions:**

The study of the use of cognitive training and psychoeducation in patients with epilepsy for cognitive functions, quality of life resulted in a positive outcome. Cognitive online training is an encouraging area in the rehabilitation of patients with cognitive decline.

**Disclosure:**

No significant relationships.

